# Is a veterinary student’s performance on multiple-mini interviews affected by personality preferences?

**DOI:** 10.5116/ijme.5c39.c815

**Published:** 2019-01-25

**Authors:** Munashe Chigerwe, Karen A. Boudreaux, Jan E. Ilkiw

**Affiliations:** 1 Department of Veterinary Medicine and Epidemiology, University of California Davis, Davis CA, USA; 2 Deans Office, University of California Davis, Davis, CA, USA; 3 Department of Surgical and Radiological Sciences, University of California Davis, Davis CA, USA

**Keywords:** Multi-mini interview, veterinary student, personality, Myers-Briggs type indicator, admission process

## Abstract

**Objectives:**

The objectives of this study were to evaluate the association between a student's Myers-Briggs Type Indicator (MBTI) preference pairs and resulting types and his or her multiple-mini interview (MMI) scores upon admission, and to determine the proportions of types among veterinary classes over five years.

**Methods:**

A cross-sectional study was conducted for data collected from 706 students admitted into the University of California Davis School of Veterinary Medicine (UCDSVM) program beginning in the fall of 2013 and ending in the fall of 2018. Data consisted of a candidate's MBTI preference pairs and types which were collected during the first week of enrollment and multiple-mini interview scores from his or her admission data.

**Results:**

A total of 706 students from 5 classes completed the MBTI survey. Multivariate analysis showed no significant association between the MBTI preference pairs of extroversion and introversion (F(_1, 697) _= 3.30, p=0.0959), sensing and intuition (F_(1, 697) _= 0.40, p=0.4395), thinking and feeling (F_(1, 697)_ = 3.59, p=0.0591), or judging and perceiving (F_(1, 697) _= 0.38, p = 0.5657) and MMI score. Analysis showed no trends (χ^2^ (60, N=706) =76.51, p=0.074) in the student's MBTI types over the 5-year period.

**Conclusions:**

The MMI score of a candidate admitted into the UCDSVM is unlikely to be affected by personality preferences. Therefore, it is unlikely that multiple-mini interview scores included in the admission process will affect the personality diversity of candidates admitted into a veterinary class.

## Introduction

Admission criteria are designed to assess and accept the best candidates who are well-prepared for a particular curriculum and are most likely to succeed in that chosen course of study. The American Veterinary Medical Association states the cost of four years of resident tuition for veterinary graduates in the graduating class of 2018 ranged from $146,636 to $304,878.^1^  In spite of these high tuition costs, individuals seeking a veterinary medical degree are not deterred as they are driven by their passion for the profession.^2^   With this in mind, the admission process is increasingly more important to identify candidates who can complete the required course of study avoiding the accumulation of debt by unsuccessful students.

Veterinary medical colleges employ various criteria to select students for admission into their program of study. These criteria include applicant academic standing, letters of recommendation, personal statements, and traditional interviews. Approximately 80% of veterinary medical colleges in North America interview applicants which traditionally includes assessment of non-cognitive skills/humanistic skills and clarification of information provided in the applicant’s written application.^3^ The five most common characteristics and skills assessed with traditional interviews include communication and interpersonal skills, maturity, interest in the practice of veterinary medicine and knowledge of the veterinary profession.^3^ In North American veterinary schools, the traditional interview is conducted by a panel of 2 to 3 faculty interviewers and lasts 20 – 45 minutes.^3^ The structure and organization of the traditional interview are low to moderate in reliability.^3^ Most of the traditional interview questions posed to the applicant assess the applicant’s general background, experiences in veterinary medicine, as well as, an applicant's strengths and weaknesses.^3^ Although some level of training is provided to most interviewers, the most common training method is by the distribution of printed material.^3^ The value of traditional interviews in the admission process, and its ability to predict academic and clinical performance, has been challenged due to concerns about reliability.^4^^-^^7^ Consequently, the MMI was developed^6^ and adopted by medical^8^ and veterinary schools^9^ in North America. The MMI demonstrated adequate reliability as part of the admission process of veterinary school candidates.^10^

The MMI is a highly structured student selection method resembling the Objective Structured Clinical Examination (OSCE).^6^ In the MMI, candidates spend a short time at a series of stations consisting of different scenario supervised by a rater who is either a faculty member, staff member or outside practicing veterinarian. The scenarios are designed to meet the requirements and goals of an individual veterinary school's program through the evaluation of a candidate’s ability to logically work through a problem or present ideas clearly where the possession of specific veterinary medical knowledge is not necessary.^6^ Each MMI station involves a task, such as, reading a scenario and answering associated questions, discussing a particular viewpoint, role-playing or completing a task. The candidate is scored on his or her critical decision-making ability, communication skills, and attitudes toward ethical and social scenarios of the profession.

The University of California Davis School of Veterinary Medicine (UCDSVM) began including the MMI as part of the admission process in the fall semester of 2013 with the incoming class that would graduate in 2017, referred to as the Class of 2017. The UCDSVM changed the admission process to include the MMI based on analysis of data from four previous admission cycles consisting of the classes of 2012 through 2015 who entered in the fall semesters of 2008 through 2011. The analysis demonstrated a poor correlation between points awarded for the personal statement (R^2^= 0.01, p= 0.053) or traditional interview (R^2^=0.0036, p = 0.465), and academic success within the curriculum. The medical literature supported the adoption of the MMI as a preferred method for student selection in healthcare professions, with MMI interview scores correlating with the performance of medical students on OSCEs.^11^^-^^12^ The UCDSVM admission process first used cognitive indicators, such as grade point average and scores on the Graduate Record Examination (GRE), to determine which candidates were selected for the MMI. The final selection of the incoming class was based solely on a candidate's performance on the MMI scenarios. Because the MMI was designed to assess non-cognitive abilities of a candidate and it was the method used to select the final class composition in the admission process, instructors (faculty, staff, and veterinarians) were concerned that the performance by a candidate on the MMI might be affected by his or her personality preference and could result in the creation of veterinary classes with homogenous personality preference profiles. Veterinary classes with homogenous personality preference profiles could potentially reduce personality diversity, attitudes, behaviors, and opportunities for a student's professional and personal growth. In order to explore this concern, the MMI score was correlated with personality preference pairs and resulting types as defined by the MBTI.^13^

The MBTI is considered one of the most popular personality inventory in the world, leading the market in psychological testing, and is taken by more than two million individuals annually.^14^ The MBTI is a lengthy self-reported, psychometric questionnaire based on Carl Jung's Theory of Psychological Types with the premise that variations in behavior are not random, but rather orderly and consistent and a result of the differences in the way an individual uses his or her perception and judgment. Jung explained that perception is the way an induvial becomes aware of things, people, events, or ideas, and judgment is the way conclusions are made about what is perceived. The MBTI seeks to describe an individual's preferences, referred to as type, and not a person's ability or traits. Because the MBTI uses dichotomies referred to as preference pairs, it is mistakenly referenced as a trait-based instrument suggesting that it is a measure of one's ability and is binary in nature.  The MBTI, in fact, is not binary nor does it measure ability as it is used to identify personality preferences along a spectrum suggesting that an individual uses his or her mind in certain ways which are comfortable, and, therefore, preferred, hence the term preference.^15^ An individual may exercise his or her comfortable or preferred preference more often. However, he or she does exercise both of the preference pairs, albeit most likely unequal.

The MBTI reflects four basic preferences which Jung proposed was the way an individual directed the use of his or her perception and judgment along with the way situations are attended to, and conclusions were drawn. From this theory, the MBTI was designed to determine an individual's preferences along four opposing preference pairs, or four dichotomies, reflecting dimensions or spectrums of normal or ordinary human behavior consisting of extraversion (E) and introversion (I), sensing (S) and intuition (N), thinking (T) and feeling (F), and judging (J) and perceiving (P). The terms used in the dichotomies are specifically defined by the MBTI and may not adhere to the usual definition or use of the word in everyday language. The preference pairs of extroversion and introversion explore preferences in the ways an individual focuses his or her attention to derive energy.

The preference of extroversion focuses attention on the outer world, such as people, things, or situations, while the preference of introversion focuses on an individual's inner world, such as ideas, information, explanations or beliefs. The sensing and intuition preference pairs explore preferences to which an individual pays attention, how information is received and the types of information an individual prefers. Individuals with a preference for sensing prefer factual, certainty and clarity in information, while individuals with a preference for intuition prefer to deal with ideas, ambiguity, and possibilities, as well as, anticipating what is not obvious.

The thinking and feeling preference pairs identify preferences in the ways an individual perceives information and formulates conclusions or makes decisions. Individuals with a preference for thinking are most comfortable with logical processes aimed at an impersonal conclusion or decision, while individuals with a preference for feeling tend to make decisions using their values within a context of how the outcome or result will impact individuals.

The judging and perceiving preference pairs identifies preferences in the way individuals approach and orient themselves to the outside world. Individuals with a preference for judging seek closure to situations and strives to reach conclusions and make decisions.  Individuals with a preference for perceiving are comfortable with having some vagueness or openness to situations and are comfortable with extensive exploration of possible alternatives which may prolong the decision-making process.

The MBTI consists of a questionnaire of items scored to determine an individual's four-letter type resulting from his or her dominant preferences in each of the four preference pairs. There are sixteen possible combinations indicating an individual's type as one of the following: ISTJ, ISFJ, INFJ, INTJ, ISTP, ISFP, INFP, INTP, ESTP, ESFP, ENFP, ENTP, ESTJ, ESFJ, ENFJ, and ENTJ. There is no benefit or importance associated with having one type over another as all types are equal and every type has value. The questionnaire has no right or wrong answers and does not compare individuals to norms. Since an individual can exercise both extremes of each preference pair, the identification of one's type offers further information he or she can use to understand the identified preferences better and maximizing these in various situations.

The MBTI has been used for a variety of purposes in numerous settings including employment, vocational, business, education, psychotherapy and medicine as a way to describe behavior. The MBTI can help an individual understand why he or she thinks and acts in certain ways based on understanding his or her natural or most comfortable behavior, as well as, identifying those behaviors that may not be preferred but are necessary for certain tasks.^16^

In an educational setting, the MBTI has been a useful tool to gain an understanding of one's learning styles, career interests, and promoting self-discovery.  In medical settings, the MBTI has been helpful in understanding an individual's decision-making, communication, and conflict styles, as well as, promoting teambuilding and identifying stressors. Due to these practical and necessary skills to prospective veterinarians, the UCDSVM uses this tool as part of its first-semester orientation for incoming first-year students in the Doctor of Veterinary Medicine degree program. Specifically, the MBTI is used to help students understand differences in preferences and how these can be optimized to work effectively and efficiently in small group settings which are critical to success in UCDSVM's directed problem-based learning curriculum.

Studies in medical students indicated that extraversion was associated with a higher MMI score,^17^ however, this association is unclear in veterinary medical students. A 2009 longitudinal study using the MBTI to determine preference pairs and the resulting types amongst veterinary students revealed a personality profile different from the published United States population norm.^18^ A general assumption had been made that veterinary students were predominantly ESTJ or ISTJ MBTI types and thus represented as task orientated, independent, decisive, fact-finders who enjoy a practical, logical approach and data analysis to make decisions with the main difference between the two as that of the extrovert and introvert personality preferences. The study further concluded that there was a significant shift away from this prototypical ESTJ and ISTJ type, culminating in a discernable heterogeneous profile for both males and females in the last four years of the study.^18^

We hypothesized that a student's type resulting from his or her identified MBTI preferences would not alter how he or she performs on the MMI assuming the scenarios were designed to investigate candidates’ attributes and behaviors. The objectives of this study were to evaluate the association between a student's MBTI preference pairs and resulting types and his or her MMI score upon admission and to determine the proportions of types among veterinary classes over 5 years.

## Methods

### Study design and sample

A nonprobability, convenient sample of 706 students admitted into the UCDSVM beginning in the fall of 2013, representing the Class of 2017, through the fall of 2017, representing the Class of 2021, were included in this study. Data for this study were collected as part of the admission process and content in the first block in which UCDSVM students enroll upon entry into the program. The study received expedited approval from the University of California Davis Institutional Review Board.

### Study procedures

Admission into the UCDSVM consisted of a process which first selected 240 students based on academic performance consisting of cognitive indicators, such as grade point average and scores on the GRE. These 240 students were then invited to participate in the MMI which was designed to select students with behaviors and attributes that the UCDSVM veterinary faculty considered important for a career as a veterinarian. The initial attributes, values, and behaviors assessed by the MMI were communication skills, critical thinking skills, knowledge of veterinary medicine, reliability, responsibility, moral and ethical reasoning, interpersonal skills (teamwork), problem-solving skills, and creativity/lateral thinking. Diversity, determination, and resilience were later added by the Associate Dean of Student Programs and the Admission Committee. Only scores on the MMI were used to offer candidates admission into the UCDSVM beginning with the highest score and proceeding down until all seats in the class were filled.

**Table 1:** Results of the general linear mixed model evaluating the association of 706 students' MBTI preferences pairs and MMI score for the UCDSVM veterinary medical classes during the fall of 2017 through the fall of 2021

Parameter	Estimate (95% CI)	F-value	p - value
Intercept	96.3280 (93.5729, 99.0831)		< 0.0001
E and I	1.9655 (-0.3488, 4.2798)	3.30	0.0959
S and N	0.9424 (-1.4497, 3.3346)	0.40	0.4395
T and F	2.2421 (-0.08679, 4.5710)	3.59	0.0591
P and J	-0.7235 (-3.1955 1.7485)	0.38	0.5657

E=Extraversion; I=Introversion; S=Sensing; N=Intuition; T=Thinking; F=Feeling; J=Judging; and P=Perceiving

### MMI instrumentation and administration

Prior to administration of the MMI, all raters were trained on the goals and administration of the MMI by a qualified trainer. The training consisted of reading written training materials, and a 2-hour face-to-face group training with practice scenarios for rating. The MMI consisted of eight to ten stations that lasted 10 minutes each. Each station had a scenario designed to meet at least one or more attributes identified by UCDSVM faculty as important to the success of a student in veterinary medicine. The candidate had three minutes to sit outside the room of each scenario allowing time for reading and contemplating a response. The candidate then entered the room and answered a series of questions or performed the requested task which was observed by the rater. A signal designated the end of time for each scenario in which the candidate exited the room, proceeded to the next scenario, and the rater then completed the ratings for that student. Scores for all scenarios were compiled into a final MMI score for each student. The students with the highest scores were offered admission until the class was filled.

**Table 2:** Proportions (%) of the 16 MBTI types for 706 students admitted into the University of California Davis classes of veterinary medicine during the fall of 2017 through the fall of 2021

MBTI type	Class of 2017	Class of 2018	Class of 2019	Class of 2020	Class of 2021	All classes
ENFJ	7.3	3.6	7.1	6.3	4.8	5.8
ENFP	6.6	5.1	5.7	2.1	7.5	5.4
ENTJ	8.8	7.2	1.4	2.1	3.4	4.5
ENTP	2.9	2.9	4.3	2.1	4.1	3.3
ESFJ	6.6	7.2	7.1	13.2	10.2	8.9
ESFP	2.2	5.1	2.9	4.2	2.0	3.3
ESTJ	6.6	15.9	10.0	6.9	10.9	10.1
ESTP	5.8	2.2	11.4	2.1	5.4	5.4
INFJ	3.6	2.2	5.7	5.6	6.1	4.7
INFP	3.6	2.9	5.7	7.6	2.7	4.5
INTJ	4.4	7.2	6.4	11.8	4.8	6.9
INTP	8.0	6.5	2.9	3.5	4.8	5.1
ISFJ	10.9	8.7	8.6	8.3	9.5	9.2
ISFP	2.9	2.2	2.9	3.5	3.4	3.0
ISTJ	15.3	18.1	13.6	16.7	15.6	15.9
ISTP	4.4	2.9	4.3	4.2	4.8	4.1
Total number of students	137	138	140	144	147	706

E=Extraversion; I=Introversion; S=Sensing; N=Intuition; T=Thinking; F=Feeling; J=Judging; and P=Perceiving A student's MBTI type is the result of the interaction among the four preference pairs resulting in the identification of one preference from each preference pair. For instance, a combination of Introversion (I), sensing (S), feeling (F) and perceiving (P) describes a MBTI preference of ISFP by a student. There was no evidence of trends (χ2 (60, N = 706) = 76.51, p = 0.074) of the MBTI types among the 5 classes.

### Myers-Briggs Type Indicator Form M instrumentation and administration

Personality preferences of the admitted students were determined using the MBTI Form M. The MBTI Form M consists of 93 items and was administered to all classes using the Skills One online portal, which is a copyrighted, commercially available product of CPP, Inc. The reliability of the MBTI Form M had been established through measures of internal consistency and test-retest reliability.  Cronbach's alpha was used to evaluate the internal consistency based on employment status (0.87-0.92), age group (0.86-0.92), ethnicity (0.83 – 0.91), and geographical region (0.81- 0.91). Test-retest reliability was established for men (0.53 to 0.93) and women (0.56 to .092). Construct validity was used to establish convergent and divergent validity of the MBTI preference pairs or dichotomies using established instruments of similar measures.^13^

Invitations to complete the MBTI inventory were sent via email with an electronic link through the Skills One online portal in June, July and August prior to the start of each school year. The email contained the link for completing the inventory along with an introduction to the MBTI Type Indicator and general guidelines suggested by the publisher for its completion. During the first week of school of the student's first semester, the students were led through a process to determine their best-fit MBTI type by an MBTI certified practitioner.  This process involved students determining the four-letter type that they think best fits them after they have been introduced to the characteristics and preferences of each type, read type descriptions, and reviewed their individual MBTI results. Determining the best fit type acknowledges that personalities are complex and no one type will adequately address all personality preferences of an individual.

### Data analysis

The normality of MMI scores was checked using the Shapiro-Wilk test. Descriptive statistics for the number of students in each class were determined. The four MBTI preference pairs namely, Extraversion (E) and Introversion (I), Sensing (S) and Intuition (N), Thinking (T) and Feeling (F), and Judging (J) and Perceiving (P) were considered. The proportion of the resulting 16 MBTI types were determined. A Chi-squared was used to examine if there were differences in the proportion of the MBTI types among the five classes. Likewise, a Chi-square (or Fisher exact test when a cell had five counts) test was used to determine if there were any trends in the 16 MBTI types among the five classes. A general linear mixed model was used to determine if the MBTI preference pairs were associated with the MMI score. In the model, the MBTI preference pairs (two levels for each of the four dichotomies) was the independent variable, whereas the MMI score was the dependent variable. Data analysis was performed using SAS version 9.4. In all analyses, p < 0.05 was considered significant.

## Results

A total of 707 students from the classes of 2017, 2018, 2019, 2020 and 2021 were invited to complete the MBTI after enrollment into the UCDSVM. A total of 706 students completed the MBTI with a response rate of 99.9 % (n = 706). The number of students from the Classes of 2017, 2018, 2019, 2020 and 2021 was 137, 138, 140, 144 and 147, respectively. No difference was found in the proportions of MBTI preference pairs among the five classes; E and I (χ^2^ (4, 379)= 4.68, p=0.3218), S and N (χ^2^(4, 421) = 1.183, p=0.2768), T and F ( χ^2^(4, 391)  = 6.10, p = 0.1922) and P and J (χ^2^(4, 466) = 5.60, p=0.2307). Multivariate analysis showed no significant association between the preference pairs of E and I (F_(1, 697)_ = 3.30, p=0.0959), S and N (F_(1, 697) _= 0.40, p=0.4395), T and F (F_(1, 697)_ = 3.59, p=0.0591), or J and P (F_(1, 697)_ = 0.38, p = 0.5657) and MMI score. The general linear mixed model evaluating the association between the MBTI preference pairs and the MMI score is summarized in Table 1. There was no evidence of (χ^2^ (60, N = 706) = 76.51, p = 0.074) trends in the 16 MBTI types among the 5 classes. The distribution of the MBTI types among the 5 classes is summarized in Table 2.

## Discussion

The significant findings in this study indicated no differences in the proportions of the MBTI preference pairs nor were trends identified in the MBTI types, among the five veterinary classes. There was no association between the MMI score and the MBTI preference pairs. The results of this study suggest that under the current admission process, selection of a veterinary medicine class based on the MMI score is unlikely to affect the personality composition of the respective class. This further suggests that the MMI does not select for one MBTI preference pair or type over another.

Specific abilities associated with the students’ personality preference include critical thinking, ethical decision-making, and interpersonal skills, all of which are assessed by the MMI.^20^^-^^25^ While there are no comparable studies in medical or veterinary medical students utilizing the MBTI preference pairs or types, some medical schools utilize the five-factor model of personality (Big Five Personality Test). This model measures personality traits of agreeableness, conscientiousness, extraversion, neuroticism, and openness. Using this model and its associated definitions for each trait, studies in medical schools suggested that extraversion was associated with a higher MMI score.^17^^,^^26^  In contrast, other studies found there was no association between a higher MMI score and personality preferences.^27^ Griffin and Wilson^26^ found a positive association between student's extraversion and conscientiousness, as identified by the Big Five Personality Test and their MMI scores, but a negative association between self-consciousness and the MMI scores. Based on the positive association, the authors reported that those who performed well on the MMI were likely to enjoy being around people, were energetic, enthusiastic, action-oriented individuals who were likely to strive hard to achieve excellence, were reliable and hard-working, and persisted even when a task was difficult or unpleasant.^26^ Kulasegaram and others,^27^ investigated the association between personality tests and the MMI to determine whether personality tests could be used early in the admission process to screen students for non-cognitive skills. Since no association was found even for conscientiousness, they determined that personality testing was not a useful screening method for the MMI.^27^ While the MBTI does not test for conscientiousness, it identifies the preference pair of intuition and sensing which is the preference of how an individual receives information and not a trait measurement of how careful and meticulous an individual is in his or her daily work. Therefore, it was not surprising that results from this study were different from those of Griffin and Wilson^21^ and Jerant and others^17^ in that extraversion and the MMI was not positively associated. A possible reason for the difference is that the scenarios in this study were designed to evaluate non-cognitive skills and not the determination of specific traits, such as an outgoing, energetic, and social person. Thus, differences in the association between the MMI score and personality preferences depend on how the MMI scenarios are written, and the abilities and behaviors that they are designed to assess, as well as, how the selected instrument defines personality. Since the MBTI determines an individual's preferences, there should be no difference in MMI task completion as no one preference is better, right, or advantageous over another.

Findings from our study further suggest that there are no preferences for a specific personality type within the student population admitted to the UCDSVM program. These results differ from a previously published study that reported on MBTI personality profiles of students admitted to Louisiana State University, School of Veterinary Medicine from 1996-2007.^18^ This 12-year composite descriptive study reported that the personality profile was different from the United States population norm, but similar to the bimodal ESTJ-ISTJ reported in medical students.^18^ A 12-year trend analysis revealed a significant shift away from the prototypical ESTJ-ISTJ profile, culminating in a discernible profile for both males and females in the last four years of the study.^18^ Differences between the study by Johnson and others^18^ and our study could be due to differences in the student populations between the two schools, as well as, the period or generation of students studied. Specifically, the applicant pool and admitted students to the UCDSVM and Louisiana State University are different, as the UCDSVM consists of mainly students from state of California, whereas, Louisiana State University admitted students from several states. Furthermore, the study by Johnson and others^18^ was conducted over a different period (1996–2007), and the period considered was longer (12 years) than the 5-year period in our study, as well as almost a decade between the studies. Variation in admissions criteria is likely to change over a longer study period compared to a shorter period, as in our study.

The practical importance of assessing the association between MBTI preference pairs and the MMI score was to ensure that the respective classes were diverse in their preferences, considering that the MMI score is used to select the admitted class. It is anticipated that a heterogeneous class with regards to personality preferences will improve the diversity of thoughts, attitudes, behaviors, and promote personal and professional growth. Also, the MBTI personality preferences and type are used to help students understand and appreciate differences between individuals, thereby facilitating and optimizing how students work in small group settings.

### Limitations

This study has several limitations. Our study population included students applying to a single veterinary school and only includes data from 5 classes. The scenarios were school-specific in that they were written by a team at the school of study and designed to assess attributes and behaviors that the faculty at the institution considered important for a successful career as a veterinarian. Furthermore, results from the MBTI are based on specific definitions for the preference pairs and types.  These definitions could vary and differ from instruments reporting to measure the same characteristic, but in fact, measure traits instead of preferences in which comparisons are not equal.  Last, the MBTI is a self-reported instrument which may be subject to lower validity from fixed choice questions, lower reliability if questions are misunderstood, social desirability bias, and acquiescence.

## Conclusions

Our study findings suggest that there are no differences in the proportions of MBTI dichotomies or trends in the MBTI personality preferences among classes or over a 5–year period in veterinary medical students. No significant association was found between MBTI personality preference and the MMI score of students admitted into veterinary school. Our study results further support that the MMI score is unlikely to influence personality preference composition of the veterinary classes suggesting that the MMI does not select for one MBTI dichotomy preference over another. Adoption of MMI as part of the admission process in veterinary colleges is unlikely to affect the diversity of thoughts, attitudes, and behaviors in veterinary medical students. There is a need for multi-institution studies examining the effect of MMI on the diversity of personality preferences, and comparison of the reliability of the association between MBTI and the MMI score compared to other personality assessment instruments such as the Big Five Personality Test Inventory.

### Acknowledgments

The authors thank Drs. Clark and Dear for administering the MBTI to the veterinary students, Dr. Crumley for training the MMI interviewers, the UCDSVM faculty, staff and outside practicing veterinarians for conducting the MMI.

### Conflict of Interest

The authors declare that they have no conflict of interest.

## References

[1] Association of American Veterinary Medical Colleges. Exploring the cost of a veterinary medical education [cited 7 Dec 2018]; Available from: http://www.aavmc.org/students-applicants-and-advisors/exploring-the-cost-of-a-veterinary-medical-education.

[2] Larkin, M. American Veterinary Medical Association. Worth the price of admission? 2015 [cited 7 Dec 2018]; Available from: https://www.avma.org/News/JAVMANews/Pages/151215b.aspx.

[3] Turnwald GH, Spafford MM, Bohr JD (2001). Veterinary school admission interviews, part 2: survey of North American schools.. J Vet Med Educ.

[4] Edwards JC, Johnson EK, Molidor JB (1990). The interview in the admission process.. Acad Med.

[5] Albanese MA, Snow MH, Skochelak SE, Huggett KN, Farrell PM (2003). Assessing personal qualities in medical school admissions.. Acad Med.

[6] Eva KW, Rosenfeld J, Reiter HI, Norman GR (2004). An admissions OSCE: the multiple mini-interview.. Med Educ.

[7] Kreiter CD, Yin P, Solow C, Brennan RL (2004). Investigating the reliability of the medical school admissions interview.. Adv Health Sci Educ Theory Pract.

[8] Eva KW, Reiter HI, Trinh K, Wasi P, Rosenfeld J, Norman GR (2009). Predictive validity of the multiple mini-interview for selecting medical trainees.. Med Educ.

[9] Hecker K, Donnon T, Fuentealba C, Hall D, Illanes O, Morck DW, Muelling C (2009). Assessment of applicants to the veterinary curriculum using a multiple mini-interview method.. J Vet Med Educ.

[10] Hecker K, Violato C (2011). A generalizability analysis of a veterinary school Multiple Mini Interview: effect of number of interviewers, type of interviewers, and number of stations.. Teach Learn Med.

[11] Eva KW, Reiter HI, Rosenfeld J, Norman GR (2004). The ability of the multiple mini-interview to predict preclerkship performance in medical school.. Acad Med.

[12] Reiter HI, Eva KW, Rosenfeld J, Norman GR (2007). Multiple mini-interviews predict clerkship and licensing examination performance.. Med Educ.

[13] Myers PB, Myers KD, Myers IB, Kirby LK. Myers-Briggs type indicator: form M. Palo Alto, CA: Consulting Psychologists Press; 1998.

[14] Arthur MB. The 'strange history' behind the Myers-Briggs type indicator- and what that can mean for you. Forbes. 2018 [cited 12 Dec 2018]; Available from: https://www.forbes.com/sites/michaelbarthur/2018/09/16/the-strange-history-behind-the-mbti-and-what-that-can-mean-for-career-owners/#3f121bd02fb3.

[15] Kerwin PL. Creating clarity: addressing misconceptions about the MBTI assessment. CPP White Paper. https://www.themyersbriggs.com/en-US/Resources/Creating-Clarity-Addressing-Misconceptions-of-MBTI.

[16] Using the MBTI in education the way it was designed. People Matters Blog: The Myers-Briggs Company, 2016 [cited 12 Dec 2018]; Available at: https://www.themyersbriggs.com/en-US/Connect-with-us/Blog/2016/December/Using-the-MBTI-in-Education-in-the-Way-It-Was-Designed.

[17] Jerant A, Griffin E, Rainwater J, Henderson M, Sousa F, Bertakis KD, Fenton JJ, Franks P (2012). Does applicant personality influence multiple mini-interview performance and medical school acceptance offers?. Acad Med.

[18] Johnson SW, Gill MS, Grenier C, Taboada J (2009). A descriptive analysis of personality and gender at the Louisiana State University School of Veterinary Medicine.. J Vet Med Educ.

[19] Schaubhut NA, Herk NA, Thompson RC. MBTI Form M manual supplement. Mountain View, CA: CPP, Inc.; 2009.

[20] Barrick MR, Mount MK (1991). The big five personality dimensions and job performance: a meta-analysis.. Pers Psychol..

[21] Clifford JS, Boufal MM, Kurtz JE (2004). Personality traits and critical thinking skills in college students: empirical tests of a two-factor theory.. Assessment.

[22] Ashton MC, Lee K (2005). Honesty-humility, the big five, and the five-factor model.. J Pers.

[23] Chamberlain TC, Catano VM, Cunningham DP (2005). Personality as a predictor of professional behavior in dental school: comparisons with dental practitioners.. J Dent Educ.

[24] Chapman BP, Duberstein PR, Epstein RM, Fiscella K, Kravitz RL (2008). Patient-centered communication during primary care visits for depressive symptoms: what is the role of physician personality?. Med Care.

[25] Bore M, Munro D, Powis D (2009). A comprehensive model for the selection of medical students.. Med Teach.

[26] Griffin B, Wilson I (2012). Associations between the big five personality factors and multiple mini-interviews.. Adv Health Sci Educ Theory Pract.

[27] Kulasegaram K, Reiter HI, Wiesner W, Hackett RD, Norman GR (2010). Non-association between Neo-5 personality tests and multiple mini-interview.. Adv Health Sci Educ Theory Pract.

